# Supplementary material for: Development of an integrated and decentralised skin health strategy to improve experiences of skin neglected tropical diseases and other skin conditions in Atwima Mponua District, Ghana
Source: PLOS Glob Public Health. 2024 Jan 19;4(1):e0002809. doi: 10.1371/journal.pgph.0002809 (PMC10798462; doi:10.1371/journal.pgph.0002809)
Supplement: S1 Text — (DOCX) [file pgph.0002809.s001.docx]

S1 Text Patient care pathways, wound management protocols and wound-care clinics

**Patient care pathways and associated wound management protocols:** These are described in detail in the accompanying tables. In brief, BU category 1 and 2 lesions, leprosy and yaws cases, wounds of other aetiology (including those with secondary bacterial infection or requiring suturing or debriding) and common skin problems including scabies, impetigo, superficial fungal infections, and ulcers requiring wound care will be diagnosed and then managed by CHPS or health centres within the district, with the patient never having to travel further for diagnostic testing or to receive medicines, supplies or care. Patients with category 3 BU lesions; wounds of equivalent size (>15cm); other wounds requiring intensive wound management or surgical treatment; signs and symptoms of a leprosy reaction and treatment resistant yaws; or other complex skin disease not manageable at peripheral health facilities will be referred to the district hospital to receive their diagnosis, start treatment (including any inpatient care required), and then to receive periodic checks. Interim care will be provided through their local health facilities, as needed. Upon clinical improvement, patients will be referred to their local health facility for all follow-up care.

Appropriate care provision is first dependent upon receiving the correct diagnosis. Across all conditions, patients will first be identified as presumptive cases when seeking care at the health facility based on signs and symptoms. Diagnosis of BU will be confirmed within a target of 7-days through molecular methods at the KCCR reference facility using swabs or FNA samples taken at health facilities; yaws will be confirmed through rapid diagnostic tests (SD-bioline and DPP) conducted at the health facility; leprosy through clinical confirmation by the disease control officer who will travel to the health facility to perform diagnosis; diagnosis of all other wounds and skin problems will follow existing practice in the district which mainly happens via presumptive diagnosis. Cases will be regularly reviewed at follow-up visits by trained clinical staff. In case of treatment failure, the reasons for treatment failure will be considered and acted upon, including alternative diagnosis or revised treatment plans.

Care costs associated with diagnosis, and with medication and wound dressings, will be covered by the study; consultation fees will be covered either by the patient or through the NHIS scheme, dependent upon enrolment status, as is standard-of-care in Ghana.

**Weekly wound-care clinics:** As a result of intervention activities, we expect to see an increasing number of patients with chronic wounds requiring regular follow up. The number of patients, the time needed to provide wound care, and limited clinic space may create a strain on health facilities. Further, clinical management protocol of wounds is shared across most aetiologies, aside from anti-microbial components, and represents a logical platform for integrated service delivery. We will therefore support health facilities to establish a weekly wound clinic voluntarily attended by patients undertaking ongoing wound management within the facility catchment population. We hypothesise that establishing dedicated clinics at every facility will support better planning for the health facility in-charge, will represent a visible and accessible point of care for affected individuals, and will facilitate the establishment of informal peer support networks amongst those receiving care and those that accompany them. Taken together this will support adherence to individualised care plans. Wound-care clinics will be open to everyone with wounds requiring specialised care, irrespective of aetiology.

Prior to the start of the intervention, the infrastructure of each health facility will be assessed to determine if it meets the requirements for adequate wound management: drinking quality water supply, wound showers, grey water and waste disposal system, examination beds and a lighting system. If these are not present, they will be provided by the project. At the weekly clinic, trained facility leads will provide information and guidance on wound management to the group of attendees based on existing guidelines and GHS protocols. Patient wounds will be examined by trained staff to assess healing and identify potential complications, for example, the emergence of secondary infections. Dressing materials will be provided as required per patient and trained staff will demonstrate and support patients unfamiliar or directly requesting assistance with dressing. Peer support will also be encouraged so that individuals with longer periods of attendance can encourage, discuss and support other patients earlier on in the care and healing pathway.

Given the variation in size and community dynamics, the exact mechanisms and delivery will vary by facility. Trained clinical leads will thus be empowered to adjust the delivery platform at their discretion, however, clinics will be mandated to conduct checks on wound progression for attendees and all activities will require oversight by formally qualified trained staff to ensure appropriate adherence to optimal care guidelines and to manage appropriate group dynamics. To ensure patient safety among those registered for wound management pathways, clinical staff will maintain a register and assess attendance at weekly wound clinics. If patients fail to attend, community-based health teams will be asked to follow-up with patients following normal GHS pathways to facilitate clinic attendance and re-supply case management materials.
