# Supplementary material for: Development of an integrated and decentralised skin health strategy to improve experiences of skin neglected tropical diseases and other skin conditions in Atwima Mponua District, Ghana
Source: PLOS Glob Public Health. 2024 Jan 19;4(1):e0002809. doi: 10.1371/journal.pgph.0002809 (PMC10798462; doi:10.1371/journal.pgph.0002809)
Supplement: S1 Table — (DOCX) [file pgph.0002809.s002.docx]

S1 Table Patient care pathways for BU

| **Condition and summary** | **Patient Contact Pathway** | **Diagnosis** | **Frontline medicines** | **Wound care** |
| --- | --- | --- | --- | --- |
| **Buruli Ulcer**  **Category 1 and 2 lesions**  ***Dual aims*** *are for:*  *(i) all patient contact points to occur at CHPS or health centres within the district (or hospital if preferred by patient); (1) patients (with support of family) will be able to manage their condition from home with bi-weekly follow up visits at local/preferred health facility.* | **First consultation**: initial care-seeking   - Identify as suspected BU based on signs and symptoms - Swab (or FNA) taken - Wound dressed, if necessary - Counselling on initial wound management and advice on diagnostic process and expected timelines   **Second consultation**: receive diagnosis and start treatment   - Patient requested to bring supportive family member or friend - Initiate treatment - Receive treatment pack (2-week supply, to take at home) - Receive training on home-based wound management and wound care pack - Receive counselling on prevention of disability (POD) activities and psychosocial support as needed   **Fortnightly follow up appointments** at wound care clinic [up to 4 months]   - Clinical check to review healing, confirm compliance - Receive treatment pack (2-week supply, to take at home) for first 8 weeks - Receive wound care pack - Receive additional advice on POD and psychosocial support as needed | - Swabs (or FNA) taken at local/preferred facility by trained PA, nurse or midwife. - all reagents and materials required for this stocked by the local/preferred facility - sample transported to reference lab (KCCR) for analysis, with transport organised by district DCO - Result communicated directly to local/preferred facility [via email] with all other levels [region, district etc] in cc. - local/preferred facility responsible for bringing patient back to facility to receive diagnosis. - Community-based health workers to follow up with patients who do not return to the facility to receive their diagnosis | - Rifampicin and clarithromycin stocked at region or district - Full treatment course transported to local/preferred facility as soon as diagnosis is confirmed by KCCR, to be coordinated by the DCO. - Health facility then package these into two-week supply packs specific to the patient - Patient visits health facility to collect medicine pack; patient to take medicines at home observed by support person (family member, CBSV) - Patient to return to local/preferred facility every fortnight to confirm they’ve been adhering and collect next pack of medicine (expected 4 follow up visits for treatment, but will continue for wound dressing) | - Wound care packs for smaller lesions stocked at the local/preferred facility. Facility personnel responsible for monitoring and ordering stocks from District. - Patient visits local/preferred facility with support person to receive diagnosis. At this appointment, training is provided to patient and caregiver on how to change dressings and manage wound at home. - Patient provided with wound care pack (dressings etc) which is intended to last two weeks. - Patient to return to local/preferred facility every fortnight for clinical check and to collect next wound care pack (expect up to 4 months of FU visits, last to confirm no further packs required) |

| **Condition and summary** | **Patient Contact Pathway** | **Diagnosis** | **Frontline medicines** | **Wound care** |
| --- | --- | --- | --- | --- |
| **Buruli Ulcer**  **Category 3 lesions**  ***Aim*** *is for travel to district hospital to be minimised, with most patient contact occurring at local health facility.* | - Patient may have to travel to district hospital for periodic checks, reducing in frequency with time. All other appointments with GHS occur at closest CHPS or health centre   **First consultation** (at health centre / CHPS): initial care-seeking   - Identify as suspected BU based on signs and symptoms - Swab (or FNA) taken - Wound dressed, if necessary - Counselling on initial wound management and advice on diagnostic process and expected timelines   **Second consultation** (at district hospital wound clinic): receive diagnosis and start treatment   - Initiate treatment - Receive treatment pack (2 week-supply, to take at home) - Wound assessed and dressed - Receive wound care packs to take to local facility for onward management   **Frequent follow up visits (every other day)** at CHPS/health centre: wound care   - Clinical check to review healing and confirm adherence to treatment. - Dressings changed using wound care packs provided by hospital - Receive counselling on prevention of disability (POD) activities and psychosocial support as needed   **Fortnightly follow up appointments** with hospital or during clinical outreach at CHPS/health centre:   - Clinical check to review healing, confirm adherence, address concerns and surgery requirements etc. - Receive treatment pack (2 weeks, to take at home) - Receive wound care packs (to take to health facility) | - Swabs (or FNA) taken at CHPS/health centre by trained PA, nurse or midwife. - all reagents required for this stocked by the CHPS / health centre - sample transported to reference lab (KCCR) for analysis, with transport organised by district DCO - Result communicated directly to CHPS / health centre [via email] with all other levels [region, district etc] in cc. - CHPS / health centre responsible for sending patient to district hospital to receive diagnosis, have clinical assessment of wound and start care - Community-based health workers to follow up with patients who do not return to the facility to receive their diagnosis | - Rifampicin and clarithromycin stocked at region or district - Patient visits district hospital to collect medicines, which are provided in 2 weekly packs; patient to take meds at home observed by support person (family member, CHSV)   *Only if fortnightly checks aren’t conducted through clinical outreach*   - Patient to return to hospital every two weeks to confirm they’ve been adhering and collect next pack of medicine | - Category 3 wound care packs stocked at the hospital. - Patient asked to attend hospital with family member to receive diagnosis. - At this appointment, patients are provided with wound care packs (dressings etc) to take with them to their local facility for dressing changes. - Patient visits CHPS / health centre every other day for clinical check and to have dressings changed   *Only if fortnightly checks aren’t conducted through clinical outreach*   - Patient to return to hospital every two weeks for clinical check and to collect next pack wound care pack |
