# Supplementary material for: Development of an integrated and decentralised skin health strategy to improve experiences of skin neglected tropical diseases and other skin conditions in Atwima Mponua District, Ghana
Source: PLOS Glob Public Health. 2024 Jan 19;4(1):e0002809. doi: 10.1371/journal.pgph.0002809 (PMC10798462; doi:10.1371/journal.pgph.0002809)
Supplement: S2 Table — (DOCX) [file pgph.0002809.s003.docx]

S2 Table Patient care pathways for Yaws

| **Condition and summary** | **Patient Contact Pathway** | **Diagnosis** | **Frontline medicines** | **Wound care** |
| --- | --- | --- | --- | --- |
| **Yaws**  ***Aim*** *is for care of yaws to be managed at the health facility level, with peripheral oversight provided by DCO* | All appointments with GHS occur at health centres or CHPS.  **First consultation**: initial care-seeking   - RDTs performed (SD Bioline & DPP) - Wound dressed, if necessary - Treatment provided (penicillin or azithromycin)   **Second consultation**: 4-week follow up   - Clinical check to review healing. - If wound hasn’t healed take lesion swab (to test for resistance) and provide penicillin (if not given first time). | - Both RDTs stocked at CHPS/health centre, administered by trained PA, nurse or midwife. - All reagents required for this stocked by the CHPS / health centre - *In context of treatment failure:* - swabs available at CHPS/health centre - sample transported to reference lab (KCCR) for analysis - result communicated directly to CHPS/health centre [via email] with all other levels [region, district etc] in copy. - if azithromycin resistance detected, CHPS/health centre is responsible for notifying patient. Ongoing management & contact tracing responsibility of DCO | - Penicillin or azithromycin stocked at the health centres/CHPS - Facility personnel responsible for monitoring and ordering stocks from District. | **If necessary (less common):**   - Wound care packs for smaller lesions stocked at the CHPS/health centre. Facility personnel responsible for monitoring and ordering stocks from District. - Patient visits CHPS/ health centre with support person to receive diagnosis. At this appointment, training is provided to patient and caregiver on how to change dressings and manage wound at home. - Patient provided with wound care pack (dressings etc) – it’s expected that only one pack will be needed |
