# Supplementary material for: Development of an integrated and decentralised skin health strategy to improve experiences of skin neglected tropical diseases and other skin conditions in Atwima Mponua District, Ghana
Source: PLOS Glob Public Health. 2024 Jan 19;4(1):e0002809. doi: 10.1371/journal.pgph.0002809 (PMC10798462; doi:10.1371/journal.pgph.0002809)
Supplement: S3 Table — (DOCX) [file pgph.0002809.s004.docx]

S3 Table Patient care pathways for Leprosy

| **Condition and summary** | **Patient Contact Pathway** | **Diagnosis** | **Frontline medicines** | **Wound care** |
| --- | --- | --- | --- | --- |
| **Leprosy**  ***Aim*** *is for care of leprosy to be managed at the health facility level, DCO visiting to confirm diagnosis and initiate treatment.* | **First consultation** at health centre or CHPS**:** initial care-seeking   - Suspected diagnosis based on signs and symptoms - Patient receives advice on confirmatory pathway - DCO notified.   **Second consultation (at health centre or CHPS within 2 weeks):** confirmation and initiating treatment   - DCO examines and confirms diagnosis. Assessment of leprosy-associated disability and determine where ongoing management for this should be [presumption – unless necessary, to be at the CHPS/health centre] - Initiate treatment and receive treatment pack (4weeks, to take at home) - Receive training on self/home-based care, if necessary - Receive advice on prevention of disability   **Monthly follow up appointments** [6 to 12 months minimum] at health facility / CHPS:   - Clinical check to review nerve function, confirm compliance, assess for reactions and new disability - Receive treatment pack (4 weeks, to take at home) - If clinical suspicion of reaction, refer for review by DCO [at CHPS/health centre]. | - Initial examination at CHPS/health centre by trained PA, nurse or midwife. - DCO confirmation at CHPS/health centre within two weeks. | - Multidrug treatment regimen stocked at region or district. - Full course of medicines transported to CHPS/health centre by DCO at the time of review consultation. | **If necessary (less common):**   - Wound care packs for smaller lesions stocked at the CHPS/health centre. Facility personnel responsible for monitoring and ordering stocks from District. - Patient visits CHPS/ health centre with support person to receive diagnosis. At this appointment, training is provided to patient and caregiver on how to change dressings and manage wound at home. - Patient provided with wound care pack (dressings etc) to last two weeks. - If necessary, follow up visits to assess whether more packs are needed. |
