# Supplementary material for: Development of an integrated and decentralised skin health strategy to improve experiences of skin neglected tropical diseases and other skin conditions in Atwima Mponua District, Ghana
Source: PLOS Glob Public Health. 2024 Jan 19;4(1):e0002809. doi: 10.1371/journal.pgph.0002809 (PMC10798462; doi:10.1371/journal.pgph.0002809)
Supplement: S4 Table — (DOCX) [file pgph.0002809.s005.docx]

S4 Table Patient care pathways for Wounds and non-BU ulcers

| **Condition and summary** | **Patient Contact Pathway** | **Diagnosis** | **Frontline medicines** | **Wound care** |
| --- | --- | --- | --- | --- |
| **Wounds and non-BU ulcers**  ***Dual aims*** *are for:*  *(i) all patient contact points to occur at CHPS or health centres within the district; (1) patients (with support of family) will be able to manage their condition from home with bi-weekly follow up visits at local health facility.* | **First consultation**: initial care-seeking   - Swab taken if indicated - Other diagnostic tests conducted, as indicated - Wound dressed, if necessary - Counselling on initial wound management and advice on diagnostic process and expected timelines   **Second consultation**: receive diagnosis (if requested) and start treatment   - Patient requested to bring supportive family member or friend - Initiate treatment (if required) - Receive treatment pack (2-week supply, to take at home) - Receive training on home-based wound management and wound care pack - Receive counselling on prevention of disability (POD) activities and psychosocial support as needed   **Fortnightly follow up appointments** at wound care clinic [up to 4 months]   - Clinical check to review healing, confirm compliance - Receive treatment pack (2-week supply, to take at home) for first 8 weeks - Receive wound care pack - Receive additional advice on POD and psychosocial support as needed | - Swabs) taken at CHPS/health centre by trained PA, nurse or midwife. - all reagents required for this stocked by the CHPS / health centre - sample transported to reference lab (KCCR) for analysis, with transport organised by district DCO - Result communicated directly to CHPS / health centre [via email] with all other levels [region, district etc] in copy. - CHPS/ health centre responsible for bringing patient back to facility to receive diagnosis. - Community-based health workers to follow up with patients who do not return to the facility to receive their diagnosis | - Antibiotics if infection is indicated - Patient visits health facility to collect medicine pack; patient to take medicines at home observed by support person (family member, CBSV) | - Wound care packs for smaller lesions stocked at the CHPS/health centre. Facility personnel responsible for monitoring and ordering stocks from District. - Patient visits CHPS / health centre with support person to receive diagnosis. At this appointment, training is provided to patient and caregiver on how to change dressings and manage wound at home. - Patient provided with wound care pack (dressings etc) which is intended to last two weeks. - Patient to return to CHPS / health centre every fortnight for clinical check and to collect next wound care pack |
