# Supplementary material for: Development of an integrated and decentralised skin health strategy to improve experiences of skin neglected tropical diseases and other skin conditions in Atwima Mponua District, Ghana
Source: PLOS Glob Public Health. 2024 Jan 19;4(1):e0002809. doi: 10.1371/journal.pgph.0002809 (PMC10798462; doi:10.1371/journal.pgph.0002809)
Supplement: S5 Table — (DOCX) [file pgph.0002809.s006.docx]

S5 Table Patient care pathways for common skin problems (Scabies, impetigo, Tinea capitis, Tinea corporis)

| **Condition** | **Patient** | **Diagnosis** | **Frontline medicines** | **Wound care etc** |
| --- | --- | --- | --- | --- |
| **Scabies**  ***Aim*** *is for patient to receive prompt diagnosis and treatment at CHPS or health centres within the district* | - All appointments with GHS occur at closest CHPS or health centre  - First consultation: initial care-seeking   - Clinical examination for diagnosis including assessing for impetigo   First line treatment provided | - No specific items required for diagnosis | - frontline medicines stocked at CHPS/health centre  (25% Benzyl benzoate lotion) | n/a |
| **Impetigo**  ***Aim*** *is for patient to receive prompt diagnosis and treatment at CHPS or health centres within the district* | All appointments with GHS occur at closest CHPS or health centre  - First consultation: initial care-seeking   - Clinical examination for diagnosis including assessing for scabies   First line treatment provided | No specific items required for diagnosis | - frontline medicines stocked at CHPS/health centre  (Flucloxacillin oral or equivalent) | n/a |
| **Tinea capitis**  ***Aim*** *is for patient to receive prompt diagnosis and treatment at CHPS or health centres within the district* | All appointments with GHS occur at closest CHPS or health centre  - First consultation: initial care-seeking   - Clinical examination for diagnosis   First line treatment provided | No specific items required for diagnosis | - frontline medicines stocked at CHPS/health centre  (Griseofulvin) | n/a |
| **Tinea corporis**  ***Aim*** *is for patient to receive prompt diagnosis and treatment at CHPS or health centres within the district* | All appointments with GHS occur at closest CHPS or health centre  - First consultation: initial care-seeking   - Clinical examination for diagnosis - First line treatment provided | No specific items required diagnosis | - frontline medicines stocked at CHPS/health centre  (Whitfield’s ointment or suitable topical antifungal such as 1% clotrimazole) | n/a |
