# Supplementary material for: Development of an integrated and decentralised skin health strategy to improve experiences of skin neglected tropical diseases and other skin conditions in Atwima Mponua District, Ghana
Source: PLOS Glob Public Health. 2024 Jan 19;4(1):e0002809. doi: 10.1371/journal.pgph.0002809 (PMC10798462; doi:10.1371/journal.pgph.0002809)
Supplement: S6 Table — (DOCX) [file pgph.0002809.s007.docx]

S6 Table Diagnostics, wound care and treatment packs provided to facilities through the interventions

|  | **Buruli Ulcer** | **Yaws** | **Leprosy** | **Other wounds and lesions requiring enhanced intervention** | **Common skin problems (Scabies, Impetigo, Superficial fungal infections)** |
| --- | --- | --- | --- | --- | --- |
| **Diagnostics materials** | *to be held at the health facility level and replaced as needed****:*** separate swabs and FNA kits containing: swab sticks, transport medium, sample collection tubes, syringe/needle, gloves, saline, gauze sponge | *to be held at the health facility level and replaced as needed****:*** Syphilis RDT, DPP, gloves, saline | *No specific diagnostics required* | *No specific diagnostics required* | *No specific diagnostics required* |
| **Drugs** | *to be held at district level and transported to facility when diagnosis is confirmed:* clarithromycin & rifampicin in two-week regimen packs | *to be held at the health facility level and replaced as needed:* azithromycin, benzathine penicillin, | *to be held at district level and transported to facility by dco at consultation:* WHO multi-drug therapy blister packs (dapsone, rifampicin & clofazimine) | *No specific drugs required* | *to be held at the health facility level and replaced as needed:* 25% Benzyl benzoate lotion, permethrin; flucloxacillin oral (or equivalent); griseofulvin; Whitfields ointment or 1% clotrimazole. |
| **Wound care** | *To be held at the health facility level and replaced as needed****:***   - Smaller wound dressing packs: these will be sufficient for facility- or home-management of category 1 and 2 type lesions to last two weeks. These will be available for the holistic management of all wounds and lesions that meet clinical criteria including a maximum diameter of <15cm and not involving a critical site such as a joint. - Packs will include cotton balls, sterile gauze, gloves, saline solution, povidone iodine, plasters and bandages   *To be held at the district hospital and provided to patients when they receive their diagnosis****:***   - Large wound dressing packs: these will be sufficient for facility- or home-management of category 3 sized lesions to last two weeks. These will be intended for all wounds and lesions that meet clinical criteria specified for large, complex wounds requiring referral such as wounds > 15 cm in size. - Packs will include cotton balls, sterile gauze, gloves, saline solution, povidone iodine, plasters and bandages | | | | |
