# Supplementary material for: Development of an integrated and decentralised skin health strategy to improve experiences of skin neglected tropical diseases and other skin conditions in Atwima Mponua District, Ghana
Source: PLOS Glob Public Health. 2024 Jan 19;4(1):e0002809. doi: 10.1371/journal.pgph.0002809 (PMC10798462; doi:10.1371/journal.pgph.0002809)
Supplement: Attachment — Submitted filename: Response to Reviewers.docx [file pgph.0002809.s009.docx]

8^th^ October 2023

Kumasi Centre for Collaborative Research

Kwame Nkrumah University of Science and Technology

Kumasi, Ghana

The Editor,

PLOS Global Public Health Journal

Dear Sir,

**Re: PGPH-D-23-01377 ‘’Development of an integrated and decentralised skin health strategy to improve experiences of skin neglected tropical diseases in Atwima Mponua District, Ghana’’**

My co-authors and I, wish to thank the editors and reviewers for the feedback on our manuscript. We have taken note of the review comments and revised the manuscript as suggested. The point-by-point response to the review comments are detailed below:

Journal Requirements:

1. Please review your reference list to ensure that it is complete and correct. If you have cited papers that have been retracted, please include the rationale for doing so in the manuscript text or remove these references and replace them with relevant current references. Any changes to the reference list should be mentioned in the rebuttal letter that accompanies your revised manuscript. If you need to cite a retracted article, indicate the article’s retracted status in the References list and also include a citation and full reference for the retraction notice.

Response: We have revised and formatted the references in accordance with the journal requirements.

2. Please provide separate figure files in .tif or .eps format only and remove any figures embedded in your manuscript file. Please also ensure all files are under our size limit of 10MB.

Response: Done as suggested. Figures 1 and 2 have been uploaded as .tiff files.

**Reviewer #1:**

Few comments

1. Reference to WHO Framework of skin NTDs is missing (https://www.who.int/publications/i/item/9789240051423). It is a companion document to the NTDs road map.

Response: We have added this.

2. Is there a role of private healthcare providers and facilities? I only see reference to GHS facilities.

Response: Although there are two private facilities in the district our intervention is focused on GHS facilities so that it can be sustainably integrated into the health system.

3. Page 7 last line: Can you please add reference to the essential medicines list from the NHIS?

Response: We have added this response.

4. Page 8 (d): Use of Whatsapp is a good and effective medium to communicate. Confidentiality is sometimes raised as an issues. What steps will be taken to protect participants confidentiality?

Response: Thank you. We have rephrased this sentence to clarify the role of WhatsApp and to confirm that there will be no use of patient identifiable information which would compromise confidentiality. The section (page 8) now reads:

‘’We will use a secure, mobile phone-based communications platform (WhatsApp) to facilitate requests for tests and medicines between levels of the health system and to alert health facility staff of positive test results reported through the usual GHS system. No patient identifiable data will be shared using this platform.”

5. Page 8 (intervention 2). Burden on families should also be mentioned especially caregivers/parents of children. Not only on the health service.

Response: Done as suggested. The sentence now reads:

‘’Self-care was proposed as a core intervention to reduce the need to travel frequently for treatment and wound dressings, and thereby reduce both the costs to patients and caregivers of receiving care and the burden on the health service.’’(page 9)

6. Page 9: For self-care of wounds, i would suggest <10 cm and not located at a site where restrictions of movement (joint or otherwise) can occur or damage to important organs.

Response: We thank the reviewer for the suggestion. From our experience working in the healthcare system in Ghana, we believe setting the wound size limit to <15cm is fair as most wounds in this size category can safely undergo home based self-care. Health workers working on skin NTDs such as Buruli ulcer (BU) already use the WHO classification schema for lesion categorization. That schema categorises BU wounds >15cm or wound locations involving joints or important structures such as eyes or genitalia as category III; these typically require special attention in the health facility setting. We believe utilising a similar approach will help to avoid any confusion in the minds of health workers when triaging and managing BU and non-BU wounds. We have however included the second suggestion on lesion location. The sentence now reads (page 9):

‘’Cutaneous ulcers manageable in community-based settings (<15cm, not involving joints or other important anatomic structures such as eyes or genitalia), or BU category I or II, leprosy-associated ulceration not requiring surgical intervention’’.

7. Page 9 (clinical review): I will suggest ...emphasize educational messages rather than promote.

Response: Done as suggested. The sentence now reads:

‘’Individuals and their wounds will be assessed at weekly wound care clinics which will provide further opportunity for health workers to address issues associated with self-care, emphasize educational messages and explore any concerns.’’ (page 9)

8. Page 9 (clinical review): I will suggest ....wounds not healing satisfactorily instead of not progressing satisfactorily.

Response: We have replaced the word ‘’progressing’’ with ‘’healing’’ as suggested (page 9)

9. Additional reference that may be useful: <https://www.ncbi.nlm.nih.gov/books/NBK553823/>

Response: This reference has been added.

**Reviewer #2: PLoS PGPH-D-23-01377 REVIEW**

Paragraph 3

As strategies are for all skin diseases and not only NTDs, the title should reflect this so the study can be seen by others to be for other skin conditions other than skin NTDs. For example: “Development of an integrated and decentralised skin health strategy to improve experiences of skin neglected tropical diseases and other skin conditions in Atwima Mponua District, Ghana”

Response: We agree and have made this change.

Introduction

The introduction was well written and in the context of the title and aim of the study. Based on World Health Organization (WHO) Neglected Tropical Diseases (NTD) Road Map for 2021–2030, global targets for the control, elimination, and eradication of 20 diseases requires development and integration of strategies or intervention that works for each country, region and district.

State some interventions (or holistic models) that have been made in similar districts within the country or other countries involving NTDs that can be a framework for a similar work by your team.

Response: We have added references to the section to address the reviewer’s suggestion as follows (page 3):

16. Lar LA, Chowdhury S, Umunnakwe C, Yahemba D, David A, Omitola OO, et al. A mixed methods evaluation of an integrated training package for skin neglected tropical diseases in Kaduna and Ogun, Nigeria. Int Health. 2023;15(Suppl 1):i75-i86. Epub 2023/03/25.

17. Chowdhury S, Adekeye O, McRae A, Olorunfemi T, Dubukumah L, Makinde O, et al. A holistic approach to well-being and neglected tropical diseases: evaluating the impact of community-led support groups in Nigeria using community-based participatory research. Int Health. 2023;15(Suppl 1):i87-i99. Epub 2023/03/25.

Stigma is a major issue in the management of skin NTDs. Clearly state steps that will be used to reduce this rather than stating education of the populace so others can use similar interventions.

Response: We agree with the reviewer that skin NTDs are associated with a significant stigma burden. However, the findings from our formative work (presented in a separate manuscript to this journal) indicate that stigma per se was not the major driver of delayed care seeking. Despite this finding our intervention is designed to address stigma by embedding respectful care into clinical practice at health facilities and utilising community and school based educational interventions.

Methods

Ethical approval was obtained and written informed consent.

Grants or sponsorship or collaborations should be expressly stated here. How is the London School of Hygiene and Tropical Medicine involved? What does this involve - Skin Health Africa Research Programme (SHARP) project in 2019? It is not clear to a reader of this study who does not know your previous works.

Response: We have included an introduction to the SHARP project in this section outlining the participating organisations. (page 4)

Does the SHARP multidisciplinary research team include a dermatologist? This should be stated if it does. If it does not, I would like to suggest that this be included in the implementation section. The involvement of a dermatologist will really be useful in training of health personnel as this work is based on a framework of providing care not just for NTDs but all other skin conditions. The WHO recognises that 10 out of the 20 NTDs have skin manifestations. This is buttressed by Figure 2 point E which was considered not feasible but training of health officials is central to addressing WHO NTDs goals.

Response: The SHARP collaboration includes dermatologists. We have clarified that the clinicians on the SHARP multidisciplinary team includes dermatologists. The section now reads:

‘’Each meeting was facilitated by the SHARP multidisciplinary research team comprising epidemiologists, clinicians (including dermatologists), health economists, and social scientists.’’ (Page 5).

The development of a conceptual ToC framework, and stakeholders ranking of the potential interventions based on their perceived impact, feasibility, and cost are two strategies worth emulating by others in developing their own interventions. The use of TIDieR (Template for Intervention Description and Replication) Checklist would ensure reproducibility and adaptability as the authors aimed for in any locality.

Response: We are grateful for the commendation.

Correct highlighted area in Figure 2 Prioritisation exercise results. There were 45 participants and not 35.

Response: Thank you. The correction has been done (see legend to figure 2 on page 6).

Collaboration and funding statement should be as clear and detailed as in the response on the manuscript draft.

Response: We have followed the guidance on funding statement as per the journal requirements.

Is there any plan for the National Institute for Health and Care Research (NIHR) to fund a reassessment of this work to assess the impact or evaluate the sustainability of the program in the future such as a 2 or 5 year plan? This can be added if there is a possibility. Sustainability of health programmes is paramount to obtaining NTDs goals.

Response: The intervention implementation includes a process monitoring and evaluation component. This is reflected in the discussion section (last paragraph) as follows: ‘’Alongside the intervention implementation, we will conduct a rigorous, multidisciplinary evaluation to assess the effectiveness, costs, and cost-effectiveness of our strategy and the processes by which the planned activities contribute – or not – to intended and unintended outcomes.’’ (page 13)

References

Correct citations. PLOS uses the reference style outlined by the International Committee of Medical Journal Editors (ICMJE), also referred to as the “Vancouver” style. Many journal titles were not abbreviated. For instance, reference 35 page 20 - “International journal of public health” should be “Int J Public Health” Reference 17 should be “BMJ” not “Bmj” as written. “PLoS Negl Trop Dis.” should replace “PLoS neglected tropical diseases” in references 31 and 32.

Response: We have updated and formatted the references in accordance with the journal style.

We thank the Editors and reviewers for the consideration given to our manuscript and look forward to the further processing of the manuscript.

Yours Sincerely,

Prof Richard Odame Phillips

Corresponding Author
