# Supplementary material for: Development of an integrated and decentralised skin health strategy to improve experiences of skin neglected tropical diseases and other skin conditions in Atwima Mponua District, Ghana
Source: PLOS Glob Public Health. 2024 Jan 19;4(1):e0002809. doi: 10.1371/journal.pgph.0002809 (PMC10798462; doi:10.1371/journal.pgph.0002809)
Supplement: Attachment — Submitted filename: 2nd Review _PLoS REVISED -PGPH-D-23-01377.docx [file pgph.0002809.s010.docx]

Review of manuscript PGPH-D-23-01377R1

Development of an integrated and decentralised skin health strategy to improve experiences of skin neglected tropical diseases and other skin conditions in Atwima Mponua District, Ghana

The authors have done a comprehensive study. The corrections of the manuscript has been diligently done save for a few references.

I have checked the each reference. It was noted that the journal names of some references were not abbreviated.

A quick way: Copy the journal name in the references to Google search bar and add the word ‘abbreviation’, this will bring out the journal abbreviation. For example, for reference number 42, ‘Annual review of public health abbreviation’ will be Annu Rev Public Health.

- Reference 9: Correction should be - Trop Med Health.
- Reference 10: Correction should be - Trop Med Int Health.
- Other references for correction of journal abbreviations to be done are reference number 20, 24, 31 (use number 40 journal abbreviation), 33, 34, 35, 36, 38, 39, 40, 41 (see below), 42.
- For reference 30, add the date cited and the web link:

[Cited _____ (input the date you used it here)] Available from: [Developing an integrated intervention manual for skin NTDs.pdf (lstmed.ac.uk)](https://countdown.lstmed.ac.uk/sites/default/files/centre/Developing%20an%20integrated%20intervention%20manual%20for%20skin%20NTDs.pdf).

- Reference 41 - Greenhalgh T, Papoutsi C. Studying complexity in health services research: desperately seeking an overdue paradigm shift. BMC Med. 2018;16(1):95. doi:10.1186/s12916-018-1089-4

The manuscript is acceptable for publishing after these corrections and may not need further review work.
