# Supplementary material for: Development of an integrated and decentralised skin health strategy to improve experiences of skin neglected tropical diseases and other skin conditions in Atwima Mponua District, Ghana
Source: PLOS Glob Public Health. 2024 Jan 19;4(1):e0002809. doi: 10.1371/journal.pgph.0002809 (PMC10798462; doi:10.1371/journal.pgph.0002809)
Supplement: Attachment — Submitted filename: Response to Reviewers.docx [file pgph.0002809.s011.docx]

30^th^ November 2023

Kumasi Centre for Collaborative Research

Kwame Nkrumah University of Science and Technology

Kumasi, Ghana

The Editor,

PLOS Global Public Health Journal

Dear Sir,

**Re: PGPH-D-23-01377R1 ‘’Development of an integrated and decentralised skin health strategy to improve experiences of skin neglected tropical diseases and other skin conditions in Atwima Mponua District, Ghana’’**

My co-authors and I, wish to thank the editors and reviewers for the feedback on our manuscript. We have taken note of the review comments and revised the manuscript as suggested.

In the point-by-point response to the review comments, the reviewer comments are in blue font and our responses are in red font.

We look forward to the publication of the accepted manuscript.

Yours Sincerely,

Prof Richard Odame Phillips

Corresponding Author

**Point-by-point response to reviewer comments**

Journal Requirements:

1. Please review your reference list to ensure that it is complete and correct. If you have cited papers that have been retracted, please include the rationale for doing so in the manuscript text, or remove these references and replace them with relevant current references. Any changes to the reference list should be mentioned in the rebuttal letter that accompanies your revised manuscript. If you need to cite a retracted article, indicate the article’s retracted status in the References list and also include a citation and full reference for the retraction notice.

Response: We have revised and formatted the references in accordance with the journal requirements.

Additional Editor Comments (if provided):

Many thanks for submitting the revised article. It is fit for publication and will be accepted, but we ask that you address the issue with the references first please.

Response: We have revised and formatted the references in accordance with the journal requirements.

Comments to the Author

Reviewer #2: Review of manuscript PGPH-D-23-01377R1

Development of an integrated and decentralised skin health strategy to improve experiences of skin neglected tropical diseases and other skin conditions in Atwima Mponua District, Ghana

The authors have done a comprehensive study. The corrections of the manuscript has been diligently done save for a few references. I have checked the each reference. It was noted that the journal names of some references were not abbreviated.

A quick way: Copy the journal name in the references to Google search bar and add the word ‘abbreviation’, this will bring out the journal abbreviation. For example, for reference number 42, ‘Annual review of public health abbreviation’ will be Annu Rev Public Health.

Comment: Reference 9: Correction should be - Trop Med Health.

Response: The correction has been done as follows:

‘’Dofitas BL, Kalim SP, Toledo CB, Richardus JH. Stigma, psychosocial and economic effects of yaws in the Philippines: an exploratory, qualitative study. Trop Med Health. 2022;50(1):1-17.’’

Comment: Reference 10: Correction should be - Trop Med Int Health.

Response: The correction has been done as follows:

‘’ Renzaho AM, Woods, P. V., Ackumey, M. M., Harvey, S. K., & Kotin, J. Community‐based study on knowledge, attitude and practice on the mode of transmission, prevention and treatment of the Buruli ulcer in Ga West District, Ghana. Trop Med Int Health. 2007;12(3):445-58.’’

Comment: Other references for correction of journal abbreviations to be done are reference number 20, 24, 31 (use number 40 journal abbreviation), 33, 34, 35, 36, 38, 39, 40, 41 (see below), 42.

Response: The corrections have been done as follows:

‘’33.	Amoussouhoui AS, Ghislain Emmanuel Sopoh, Anita Carolle Wadagni, Roch Christian Johnson, Paulin Aoulou, Inès Elvire Agbo, Jean-Gabin Houezo, Micah Boyer, and Mark Nichter. Implementation of a decentralized community-based treatment program to improve the management of Buruli ulcer in the Ouinhi district of Benin, West Africa. PLoS Negl Trop Dis. 2018;12(3):e0006291.

34.	Evans M, Phillips R, Etuaful S, Amofah G, Adomako J, Adjei O, et al. An outreach education and treatment project in Ghana for the early stage of Mycobacterium ulcerans disease. Trans R Soc Trop Med Hyg. 2003;97(2):159-60.

35.	Velink A, Woolley, R.J., Phillips, R.O., Abass, K.M., van der Werf, T.S., Agumah, E., de Zeeuw, J., Klis, S. and Stienstra, Y. Former Buruli Ulcer Patients’ Experiences and Wishes May Serve as a Guide to Further Improve Buruli Ulcer Management. PLoS Negl Trop Dis. 2016;10(12):e0005261.

36.	Boock AU, Awah, P. K., Mou, F., & Nichter, M. Yaws resurgence in Bankim, Cameroon: The relative effectiveness of different means of detection in rural communities. PLoS Negl Trop Dis. 2017;11(5):e0005557.

37.	Skivington K, Matthews L, Simpson SA, Craig P, Baird J, Blazeby JM, et al. Framework for the development and evaluation of complex interventions: gap analysis, workshop and consultation-informed update. Health Technol Assess. 2021 Sep;25(57):1-132. doi: 10.3310/hta25570.

38.	Schloemer T, Schröder-Bäck P. Criteria for evaluating transferability of health interventions: a systematic review and thematic synthesis. Implement Sci. 2018 Jun 26;13(1):88. doi: 10.1186/s13012-018-0751-8.

39.	Villeval M, Bidault E, Shoveller J, Alias F, Basson J-C, Frasse C, et al. Enabling the transferability of complex interventions: exploring the combination of an intervention’s key functions and implementation. Int J Public Health. 2016 Dec;61(9):1031-1038. doi: 10.1007/s00038-016-0809-9.

40.	Prochazka M, Timothy J, Pullan R, Kollie K, Rogers E, Wright A, et al. "Buruli ulcer and leprosy, they are intertwined": Patient experiences of integrated case management of skin neglected tropical diseases in Liberia. PLoS Negl Trop Dis. 2020;14(2):e0008030. Epub 2020/02/06. doi: 10.1371/journal.pntd.0008030. PubMed PMID: 32023242; PubMed Central PMCID: PMCPMC7001903.

41.	Greenhalgh T, Papoutsi C. Studying complexity in health services research: desperately seeking an overdue paradigm shift. BMC Med. 2018 Jun 20;16(1):95. doi: 10.1186/s12916-018-1089-4.

42.	Hawe P. Lessons from complex interventions to improve health. Annu Rev Public Health. 2015 Mar 18; 36:307-23. doi: 10.1146/annurev-publhealth-031912-114421.’’

Comment: For reference 30, add the date cited and the web link:

[Cited _____ (input the date you used it here)] Available from: Developing an integrated intervention manual for skin NTDs.pdf (lstmed.ac.uk).

Response: Done as suggested.

Comment: Reference 41 - Greenhalgh T, Papoutsi C. Studying complexity in health services research: desperately seeking an overdue paradigm shift. BMC Med. 2018;16(1):95. doi:10.1186/s12916-018-1089-4

Response: The reference has been reformatted as suggested and now reads:

‘’41.	Greenhalgh T, Papoutsi C. Studying complexity in health services research: desperately seeking an overdue paradigm shift. BMC Med. 2018 Jun 20;16(1):95. doi: 10.1186/s12916-018-1089-4.’’

The manuscript is acceptable for publishing after these corrections and may not need further review work.

Response: We thank the reviewer for the useful comments on the manuscript.
